# Perspectives on Genetic and Environmental Factors in Myopia, Its Prediction, and the Future Direction of Research

**DOI:** 10.1167/iovs.66.7.4

**Published:** 2025-06-05

**Authors:** Katie M. Williams, Christopher J. Hammond

**Affiliations:** 1 Section of Academic Ophthalmology, School of Life Course and Population Sciences, Faculty of Life Sciences & Medicine, King's College London, London, United Kingdom; 2 Moorfields Eye Hospital NHS Foundation Trust, London, United Kingdom; 3 Great Ormond Street Hospital for Children NHS Foundation Trust, London, United Kingdom

**Keywords:** myopia, genetics, environment

## Abstract

The dramatic rise in myopia prevalence over the last century is most likely a function of modern-day childhood – a reduction in time spent outdoors and increasing time on near tasks. The widespread use of handheld digital devices, especially in young children, is of concern – both in terms of myopia risk but additionally that excessive use may be linked to sociodemographic factors and could more widely negatively affect health outcomes. Refractive error is a highly heritable trait, and genetic factors are the leading determinant of refractive status variation within a particular environment. Better understanding of these genetic factors could enable prediction of future myopia status, provide novel therapeutic avenues, and personalised treatment. Monitoring axial length growth of increasing interest, likely offering better identification of pre-myopia status and a more accurate correlate of risk of future visual complications. Prediction models are increasing in utility – comprising the aforementioned factors and artificial intelligence within this area is likely to increase. Population-based interventions, such as increased time outdoors, to reduce the incidence and/or slow myopia progression have shown some success, and combined approaches hold future promise. Children developing high myopia at a young age are most at risk of future complications, yet to date are a under researched cohort. Likewise, progression and potential modification of risk in young adults requires more research. The adoption of improved technology into this field to better quantify outdoor exposure and near activities alongside ocular growth, choroidal thickness and peripheral refractive changes in all mentioned cohorts is needed.

##  

Over the last century, myopia has risen to reach epidemic proportions and is a growing health concern globally.^1^^,^^2^ The highest prevalence rates are seen in children and young adults of Chinese ancestry in East and Southeast Asia – up to 50% of 9 year old children in Singapore, 61% of 12 year old children, and 95% of university students in Taiwan, 94.9% of junior high school students in Japan, and over 96% of 19 year old men in Seoul are now affected.^1^^,^^3^^–^^6^ In the West, the figures are not as dramatic, but it appears to be similarly increasing – nearly half of 25 to 29 year old people are myopic in Europe and the rate has doubled in those born in the 1960s compared with those in the 1920s.^2^ It is predicted that 50% of the world's population will be myopic by the year 2050.^7^

Whereas genetic background is important in predicting the risk of myopia, genes cannot explain the recent epidemic. Risk factors include higher education, prolonged near work, living in urban environments, and lack of time spent outdoors. But can these associations explain why myopia is becoming more common? It could be argued that myopia is an evolutionary adaptation to a modern urbanized lifestyle involving prolonged computer work, intense education, and less time outdoors. However, evolutionary adaptation happens over a much longer time frame.

### Life-Course Epidemiology of Myopia: A Complex Trait

Contributing determinants of myopia risk vary and interact over childhood development. The life course epidemiology approach enables identification of factors contributing to myopia risk operating across these different life stages (preconception, pre-school, childhood, and adolescence), appreciation of accumulated risk, and appropriate weighting of environmental exposures during critical periods, and, in this setting, ocular growth. Using this method, studies have observed that trajectories for ocular growth appear to be influenced by determinants at birth (socioeconomic status [SES], maternal age, and maternal education level) and across childhood (education, cognition, and outdoor activity) – with the suggestion that changing sociological and lifestyle factors in childhood, such as older, more educated mothers, and early schooling, and possibly screen use, are influential.^8^^,^^9^ Knowing which life stages are most influential on ocular growth trajectories is important to understand when best to use the myopia prevention strategies.

## Environmental Risk Factors

### Outdoor Activity

Outdoor activity has been identified as one of the major factors influencing the risk of myopia. After important early studies laid the ground, the Sydney Myopia Study in particular emphasized the importance of outdoor activity (sport or leisure) not only influencing the risk of myopia but also mitigating the effects of high reading scores.^10^ The majority of epidemiological studies since have confirmed this, bar a small number.^11^^,^^12^ Meta-analysis showed highly significantly reduced risk ratios for clinical trials, longitudinal cohort studies (relative risk = 0.57) and, less strong, cross-sectional studies, all the more impressive in that most studies used parental report rather than objective measures.^13^ Seasonal changes in eye growth and animal models of myopia have confirmed the protective effect of light levels.^14^^,^^15^

With this knowledge, further research is needed with objective measurements to see if spectral composition of light is important,^16^ for example, violet light has been proposed to slow progression,^17^ and whether modulation of indoor light can truly affect myopic progression, or at least its incidence,^18^ and the exact mechanisms whereby outdoor/light inhibits eye growth.

### Education

There is a large body of work over several hundred years linking education to myopia, both in terms of the number of years of education in adult studies and in educational attainment in children studies, even trying to adjust for the number of hours of reading.^19^^–^^21^ The intensification of education systems in urban East Asia may well be part of why the “epidemic” of myopia developed there.^22^ The strength of the epidemiological associations suggests a causal link, and, as detailed below, Mendelian randomization studies have shown causality.^23^ The mechanism is unclear, and close work and reading have been implicated, as well as less time outdoors.

### Reading: Just How Good Is the Evidence?

An association between various measures of reading and myopia has been long been referenced in the literature – including self-reported prolonged periods spent reading,^24^^,^^25^ quantifiable periods of multiple near work activities in combination measured in “diopter hours,”^26^ and children reporting that they read multiple books per week.^27^ A related variable is the “liking” of reading, unless one argues that it represents a more complicated behavioral phenotype, and this too has been associated with myopia.^20^ However, the association between myopia and near work is often weak and variable in epidemiological studies, inclusive of Asian, American, and British populations.^5^^,^^28^^–^^31^ In the Sydney Myopia Study, there was similarly a weak correlation between spherical equivalent and near work activities (*r* ≤ 0.2),^32^ but a stronger relationship between myopia and intense near work – a close reading distance (< 30 cm) and continuous reading (> 30 minutes) independently increased the odds of myopia in their sample. The World Health Organization (WHO) have now sponsored the development of a simpler questionnaire to be used in epidemiological studies by adopting a diary-like format.^33^

The association between reading and myopia is probably real as it remains often significant despite use of relatively poor measures in epidemiological studies and adjusting for other confounders, such as time spent outdoors. Careful measures, including length of uninterrupted reading, reading distance, and ambient light, might provide better quantification of this risk factor.

### Do Screens Really Play a Role in the Myopia Epidemic?

Screen use has been postulated as a risk factor for myopia, given its rise in use in recent decades. Most studies have relied on questionnaires which include separate reading, computer use, and outdoor activity assessments, and these are difficult to disentangle, in addition to consideration of screen size and distance (e.g. computer monitor versus smartphone). Older studies did not show a definite association^34^ but a more recent meta-analysis suggested smartphone use on its own, and combined with computer use, is a significant risk factor.^35^ A Dutch study of teenagers with a smartphone app which monitored (average 3.7 hours/day) use showed a weak cross-sectional association with myopia, but the longitudinal Generation R study of children aged 9 years did find screen use an independent risk.^36^ The Ireland Eye Study identified an alarming rate of myopia in 15.5% of 6 year old children who used screens for more than 3 hours a day compared to 3.0% in those who used one for less than an hour (albeit outdoor time was not included in multivariate analyses).^37^ Data from the extreme environmental changes associated with coronavirus disease 2019 (COVID-19; described later in this report) were associated with significant increases in myopia prevalence, particularly in young children aged 6 to 8 years, albeit screen use was not significantly associated with incidence of myopia in a short 8-month follow-up study.^38^ Our view is that although the evidence is not strong, it would seem wise to limit screen (particularly smartphone/tablet) use in young children. Clearly, objective studies are required with real-time monitoring of screen use including overall time, working distance, uninterrupted use times, as well as other intriguing possibilities, such as the work by Schaeffel et al. suggesting that black text on white background may be myopigenic, whereas white text on black background may inhibit myopia.^39^

### Socioeconomic Status and Obesity: A New Epidemic?

Historically, almost all studies have shown that myopia is associated with higher parental education and SES, and people with myopia have lower body mass index (BMI) than people without myopia. This may be changing. For example, Israeli army conscript studies of over 100,000 19-year-old recruits found no association between BMI and myopia in 1995,^40^ whereas a 2022 study of over a million recruits showed a J-shaped curve.^41^ Previous Korean army recruit studies found that myopia is associated with lower BMI, but a recent KHANES study of over 1000 Korean children found highly myopic children more likely to be obese.^42^ Obesity in children is related to lower SES in all studies, and the rise in obesity (likely due to sedentary indoor lifestyle) may now be associated with a rise in myopia in these groups. In the Rotterdam Generation R study, 6 year old children from socioeconomically disadvantaged groups were more likely to be myopic, due to lifestyle factors.^43^ The relationship between children's screen use and SES is complex and changing, but most studies of young children (below age 9) find children of low SES spend more time on screens, although, as children get older, the differences are less but higher SES children spend more time in active educational screen work, whereas lower SES children may spend more passive time watching videos. More work is needed to study whether SES, obesity, and screen use are all proxies of lack of time outdoors, or whether they are truly independent risks for myopia. The same is true of ethnicity as a risk factor, it is likely this is a proxy of SES, cultural behaviors, and attitudes to physical exercise and schooling.

### Other Risk Factors

Many other risk factors have been suggested for myopia, with some conflicting results. These include access to green spaces, sleep quantity and quality, diet, season of birth and birth order, maternal smoking, urbanization, and pollution.^8^^,^^44^^,^^45^ There is a challenge in establishing whether they are truly independent or are confounded by the major effects of outdoor activity and education. The IMI Risk Factor for Myopia report on the epidemiology of myopia elegantly summarizes many of these aspects.^33^

## Genetics of Myopia

### Heritability: What Does it Mean in a Myopia Epidemic?

Given the rapid changes in the prevalence of myopia in the last 50 years across most populations across the world, and the slow genetic drift of evolution across many generations, it seems counterintuitive that genetic factors are important in refractive error. Family history remains the strongest predictor in epidemiological studies. Twin studies, described as nature's experiment to separate nature from nurture, are designed to reduce the effects of shared environmental factors and cohort effects of recent changes in environmental risk factors (important for myopia) in familial aggregation of disease. They report high heritability of refractive error of around 80% across many different age groups and geographic locations. A meta-analysis of twin studies found a heritability of spherical equivalent and axial length to be around 71%.^46^ It is important to understand that heritability explains the proportion of population variation explained by genetic factors, not the population mean, so a changing environment can push the population distribution to the left, to the myopic side.

However, genome-wide association study (GWAS) data find that the single nucleotide polymorphism (SNP)-based heritability is only around 20%,^47^ a paradox seen in many heritable common diseases. Our simulations suggest the GWAS SNP-heritability will plateau at around 30% when studies include 2.5 million sample sizes. This “missing heritability” may be in part due to the fact that GWAS examine additive effects of common genetic variants, and that genetic variation in refractive error may also be caused by individual rare mutations or structural alterations, such as copy number variants, by epigenetic effects, or non-additive genetic effects (e.g. gene-gene interactions), and by difference in genetic architecture between different ancestral groups.

### Genetics of Myopia

Other than genes transmitted through Mendelian inheritance (e.g. Stickler's syndrome, see the High Myopia section), little was known about the genetic factors influencing common or simple myopia in the first decade of the 21st century. There were many loci identified through family-based linkage studies but by 2010 no genes had been convincingly identified. By then, with the costs of genotyping falling, it was possible to perform GWAS studies for a variety of traits in population-based studies, and the first 2 refractive error GWAS were published in 2010 including approximately 5000 participants in each. Subsequent studies increased sample size so that by 2021 the largest GWAS (in European participants) of over half a million participants identified 449 loci at genome-wide significance influencing refractive error, all of small effect size (unlike the top loci for age-related macular degeneration, for example).^47^

Many of these genes identified, when mutated, cause Mendelian disease of almost all tissues of the eye, and there is a strong representation of retinal genes, unsurprisingly, given the role of the retina in sensing defocus and driving growth of the eye.^48^ Other genes have been associated with circadian rhythm, cognition, intraocular pressure (IOP), and this plethora of genes underlines the complexity of emmetropization and eye growth, as well as presumably many mechanisms to maintain normal eye growth given the importance of vision in human evolution.

### Gene-Environment Interactions

One of the “holy grails” of genetic epidemiology is to discover whether particular genetic variants interact with specific environmental influences to cause myopia. This has proved difficult, in part because the effect sizes of the many individual associated genetic variants are so small that generating significant statistical results is difficult. To date, some interactions have been identified between education and specific genetic risk variants, for example, those in the GJD2 gene in Dutch populations,^49^ and ZMAT4 and near work time in European childrens’ cohorts,^50^ but there is little replication and unreliable quantitative measures of other exposures, such as outdoor time, mean that this field is under-explored.

### Implications of Genetic Findings

It is a valid question to ask how these genetic results might be used in the field of myopia in the future. GWAS disease-associated SNPs are almost all in non-coding parts of the human genome, and so unlike Mendelian mutations which can be induced in animal models and explored, these loci may be involved in regulation of genes and their expression or may just be markers of disease-causing mutations nearby or elsewhere, so they are difficult to study. One hope is that new mechanisms and pathways might be identified to lead to new treatments, as has happened with the complement system and age-related macular degeneration. Further bioinformatics and wet-laboratory works are required to identify potential targets and mechanisms of action.

Genetic and bioinformatics analysis tools may be used to understand disease associations. As an example, Mendelian Randomization (MR) uses genetic SNPs identified in GWAS to examine direction of causality. Risk alleles are allocated randomly from parental genotypes at inception, akin to a randomized controlled trial, and as long as they are predictors of an (environmental) exposure and are not associated with the disease or trait themselves or other potential confounders, they can be used as instruments to infer causality. As an example of this technique, education was shown to be causative for myopia (but not intelligence),^23^ and IOP is causative of glaucoma and myopia, rather than myopia causing glaucoma as is commonly assumed.^47^ Reducing IOP might be part of myopia control strategies in the future.

Other uses of these data are in risk prediction using genetic risk scores, to assess the risk of disease, risk of progression, or even as a screening tool. This is discussed in the Prediction of Myopia section. Pharmacogenomics is a field that is steadily increasing, and genetic information regarding response or side-effects is helpful in as many as a hundred drugs to date, albeit none for eye disease. A future use might be to predict whether, for example, a child might respond to optical treatments better than pharmacological agents.

Future large studies are needed in children with collection of longitudinal data, and collection of epidemiological risk factor data as well as other Omics datasets, such as epigenetic markers, which may be a way in which environmental exposures cause disease. Identification of biomarkers using systemic omics (e.g. metabolomics and microbiome) may add to the understanding of the pathophysiology of myopia, as has been successfully shown in other fields, for example, age-related macular degeneration.^51^ However, there is tissue specificity in some processes (e.g. gene expression and epigenetics) which may limit the use of systemic biomarkers for a purely ocular disease such as myopia.

## Prediction of Myopia

Prediction of children at risk of myopia (“pre-myopia”) might allow personalized preventive measures, while the eye's natural emmetropization mechanisms are still active and control eye growth, before the ocular homeostatic mechanisms are lost. Epidemiology studies provide some evidence of risk factors and prediction.

### Family History

**Figure 1.**
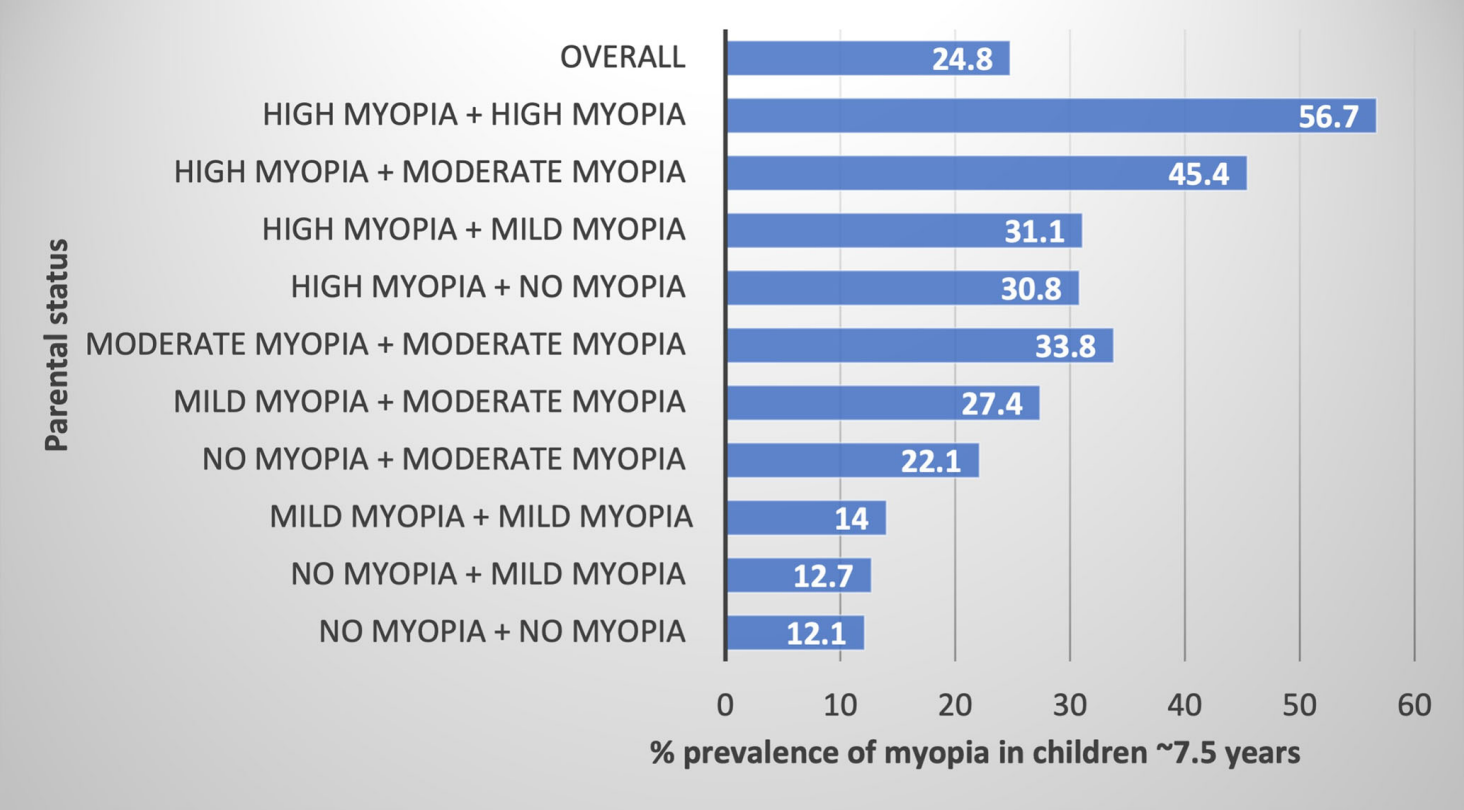
Prevalence of myopia in children aged approximately 7.5 years stratified by parental myopia status (adapted from Ref. 52).

Given how highly heritable myopia is, and the fact that the majority of epidemiological studies identify family history as the most important risk factor, a family history, and in particular one of high myopia, might allow personalized intervention for those at risk. The Hong Kong Children's Eye Study measured the actual refractive error in both parents and in over 2000 children aged 6 to 8 years and found a strong dose effect (Fig. 1).^52^ In a community setting with high levels of myopia, the highest in the world of around a quarter of children aged 6 to 8 years already affected, they showed that that 12% to 14% were affected if one or both parents were not myopic or only mildly myopic, rising to around 30% if both parents were moderately myopic or one parent was highly myopic, and, if both parents were highly myopic, 56% of their children were already myopic and almost certainly destined for high myopia.

### Cycloplegic Refraction (Less Than 0.75 Diopter Hyperopic at Age 6 Years)

It has been known for many decades that children who will go on to become myopic are already on a trajectory and that they are less hyperopic than their counterparts prior to myopia onset and that the fastest progression is as they become myopic. The classic study from the Orinda Longitudinal Study measured refractive error in 554 children in grade 3 and found a cycloplegic refraction of less than +0.75 diopter (D) hyperopic at that age was the strongest predictor of future myopia, with an area under the receiver operator curve (AUROC) of 0.88.^53^ Although measurement is commonly and (at the age of 6 years) relatively easily performed by pediatric ophthalmologists and optometrists, it does involve an intervention of cycloplegic drops which are not always fully effective, particularly in children with dark brown eyes, which can result in a false positive diagnosis of pre-myopia.

### Axial Length Growth Charts

**Figure 2.**
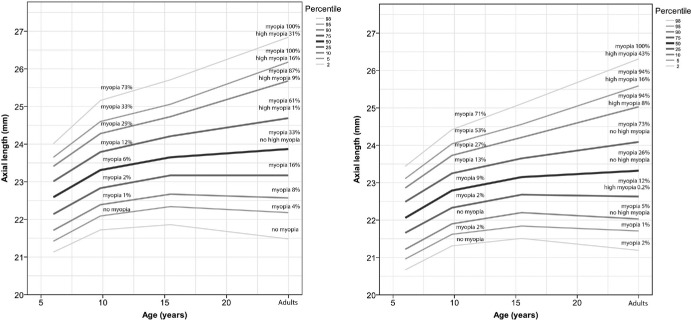
Axial length growth charts by age for girls and boys in European individuals with prediction of future myopic status – adapted from Ref. 54.

Centile axial length growth charts, akin to the widely adopted WHO children's growth curves, have been proposed over several years, and there are now several published. The Brien Holden Vision Institute's (BHVI) “Myopia Calculator” published freely available normative values of refractive error and now includes axial length (https://bhvi.org/myopia-calculator-resources/). Other groups in the Netherlands, China, and the United Kingdom have published their axial length growth curves. These publications underline the fact that axial length is also related to height, so boys tend to have longer axial lengths and so separate curves are required. The WHO publishes a single growth chart for all children “under optimal environmental conditions” rather than population-specific curves, and clearly there are huge differences between, for example, the 50% centile in European and Chinese children, although the differences at younger ages (up to approximately 6 years of age) are small. The future risk of myopia in a European population is detailed from cohort studies in the Netherlands in Figure 2.^54^ It must be emphasized, however, that the measurements are based on data taken at three time points in childhood in the Generation R Study, and in a different and much older adult cohort from two or three generations before, the Rotterdam Study.

### Genetic Prediction

**Figure 3.**
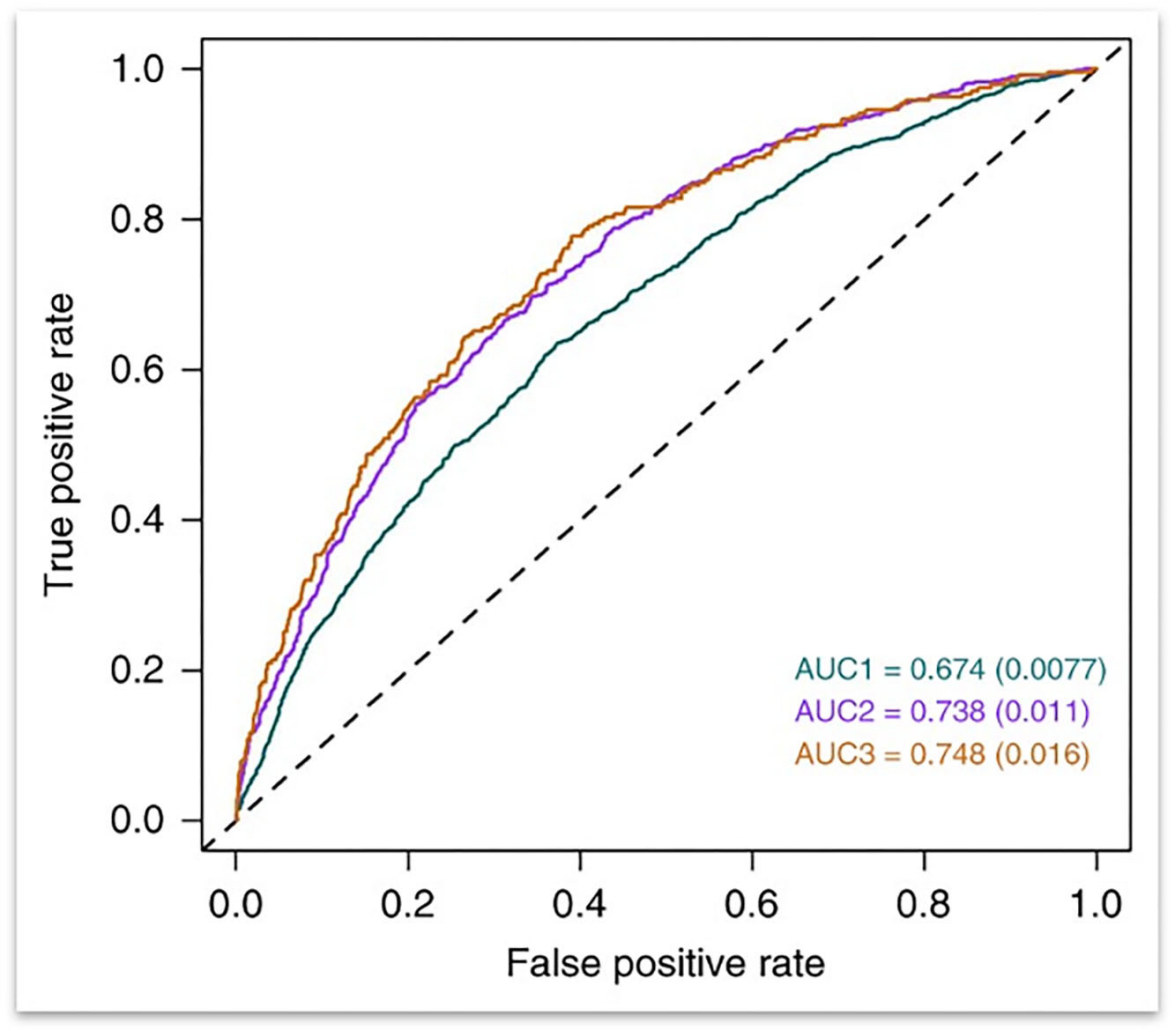
The area under the curve (AUC) predictions for low, moderate, and high myopia, from Hysi et al.^47^ The three different colors represent three different curves for each of the different definitions of myopia: *green* = all myopia (defined as <−0.75 D); *magenta* = moderate myopia (<−3.00 D); and *brown* = high myopia (<−5.00 D).

As detailed earlier, considerable advances have been made in understanding the genetics of refractive error in the population using GWAS studies, and, akin to height, it appears that there are many genetic variants of small effect adding to the risk. Prediction of myopia, particularly high myopia, is now possible using polygenic or genetic risk scores, which sum the number of risk alleles multiplied by the effect size at each for an individual. The AUC of the Hysi et al. GWAS was 0.67 for low myopia and 0.75 for high myopia (Fig. 3),^47^ whereas a more recent polygenic risk score using combined GWAS studies achieved an AUC of 0.78 for prediction of high myopia in individuals of European ancestry.^55^ However, there are considerable caveats as most GWAS to date are largely based on European populations, and the more complex genetic architecture of African populations means that predictions are significantly worse in these groups. Some progress has been made in terms of statistical modeling to improve prediction in, for example, Asian and Indian populations,^56^^–^^59^ and the National Institutes of Health have several programs underway to increase the numbers of African and African American participants in the studies, but there is still some way to go.

### Artificial Intelligence/Machine Learning

Ophthalmic imaging is readily available and an essential component to management of many retinal disorders. A study of retinal photographs in the Singapore SCORM study predicted with a high degree of accuracy which children would become highly myopic by the time they were in their late teens and therefore might benefit from myopia control treatments.^60^ Thirty percent of these children were already myopic by the age of 6 years and many of these children went on to become highly myopic, so further research is needed in this area particularly to different populations with less myopia. Artificial intelligence (AI)-derived measures from optical coherence tomography (OCT) scanning may also provide future predictions, for example, using choroidal thickness or even ocular volume as a biomarker.

### Myopia Prediction Calculators

Data-driven prediction is already a reality; in addition to the BHVI myopia calculator to predict myopia and its progression, there are also commercial prediction tools, such as the Ocumetra system which uses machine learning and combines real-life datasets with ocular factors, such as the ocular biometry and refraction, age, family history, and sex. Similarly, the non-commercial PREdicting Myopia Onset (PreMO and progression) app is designed to aid prediction and monitoring of myopia progression, as well as collect real-time data to improve understanding of the response to myopia control treatments.

The future of myopia prediction is likely to be data-driven, and may include demographic data, information on refraction, and biometry, and axial length, in particular, image analysis, and even genetic data, using machine learning tools.^61^

## High Myopia: A Neglected Subgroup

**Figure 4.**
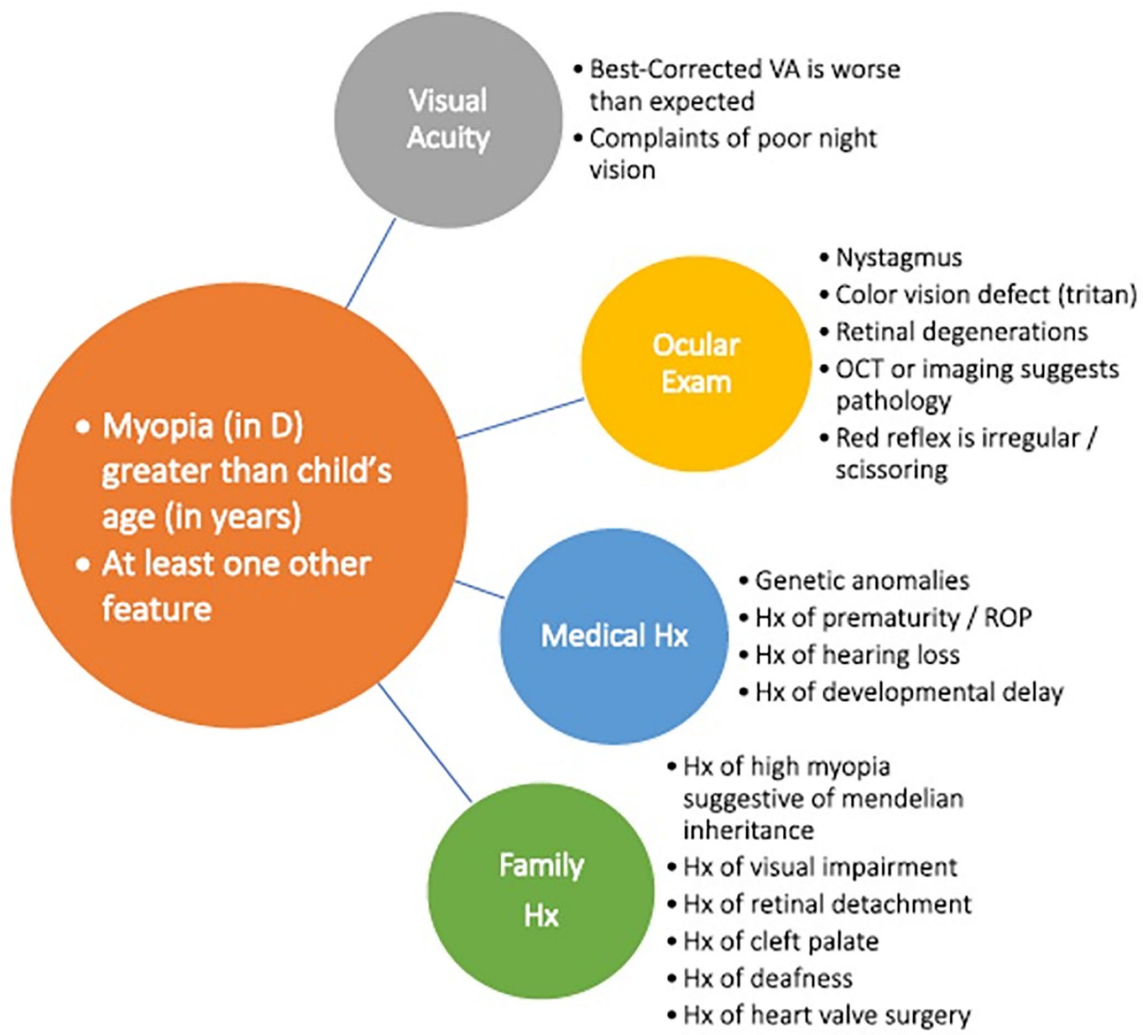
Guide for work-up of suspected cases of syndromic or secondary myopia – adapted from Ref. 67.

Fifty percent of the world's population will be myopic by 2050 and at least 10% will be highly myopic (–6 D or worse).^7^ This is concerning, as despite optical correction, myopia increases the risk of sight-threatening conditions and risk increases exponentially with increasing myopia^62^^,^^63^ – 1 in 3 patients with high myopia will develop severe visual impairment by the age of 85 years,^64^ whereas in another study, 75% of those greater than 85 years old had myopic macular degeneration.^65^ Individuals with refractive errors of −12 D or greater have a >90% lifetime risk of visual impairment.^64^ Myopia onset and progression occurs during childhood, most commonly due to axial elongation but with diverse underlying etiologies, and young age of onset is a key determinant of high myopia in later life.^54^^,^^66^ In children with early onset myopia, defined by the International Myopia Institute (IMI) as myopia in D greater than the child's age in years, further examination to explore secondary or syndromic myopia should be considered. Guidance for this was developed by the IMI^67^ (illustrated in Fig. 4).

**Figure 5.**
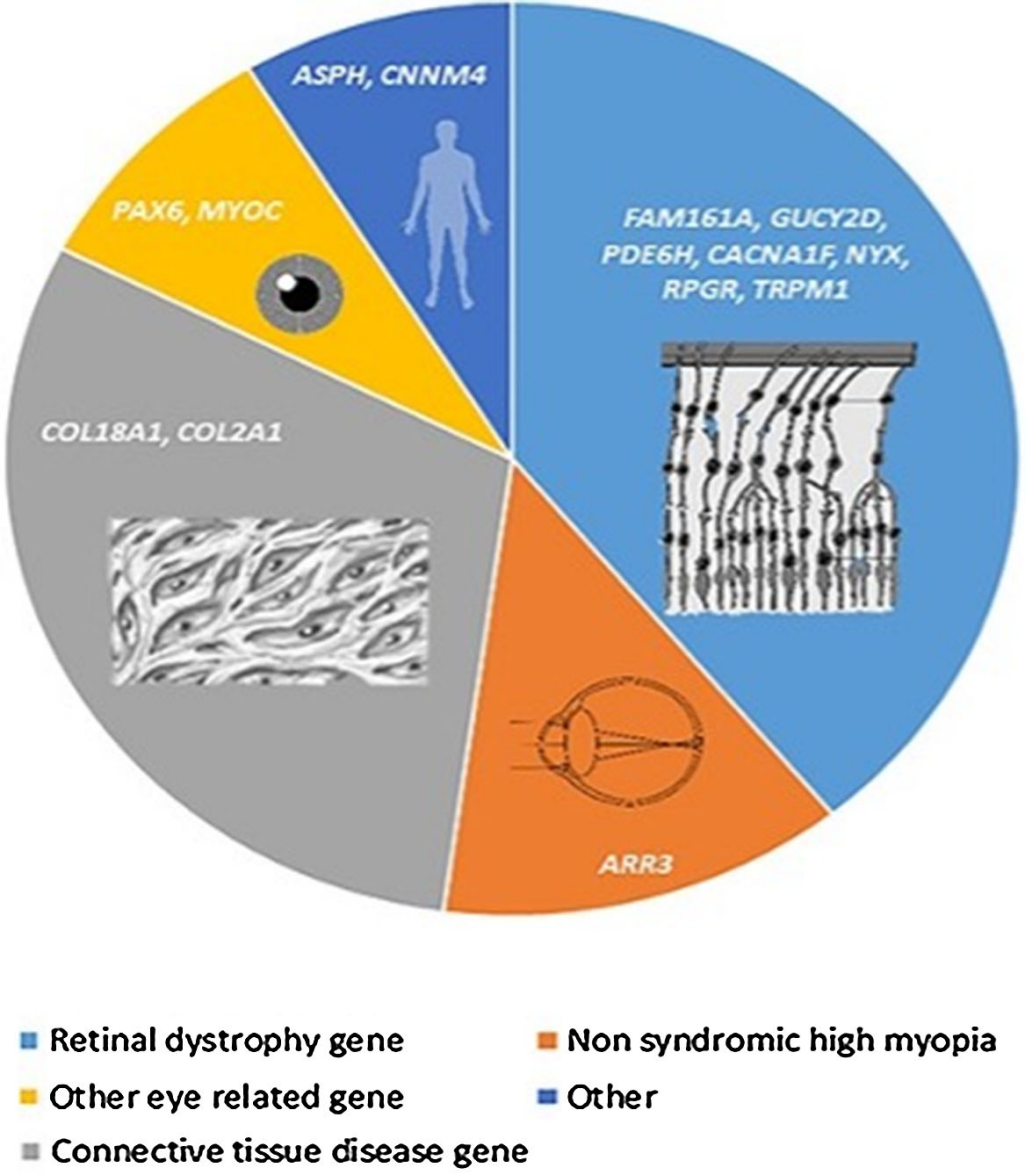
Overview of Mendelian causes of myopia – adapted from Ref. 68. Of the 23 patients with pathogenic variants – 39.1% were in a retinal dystrophy gene, 30.4% were in a connective tissue disease gene, 13.0%, in a non-syndromic high myopia gene, 8.7% in another gene, and 8.7% in other eye-related genes.

Children with early onset myopia are likely to have a stronger genetic etiology. Genetically there are two forms of myopia – those that have “common” myopia that is the result of many common genetic variants that collectively increase the risk myopia, which can be further manipulated by a number of environmental and lifestyle factors.^47^ Conversely, in Mendelian or syndromic myopia, the condition results from a single pathogenic variant, often accompanied by systemic features. Some of the common forms of Mendelian or syndromic myopia are listed Figure 5 below – with retinal dystrophy and connective disorder genes featuring most prominently in the referenced series.^68^ Mendelian causes of isolated high myopia have also been identified – including the gene *ARR3*, which appears to be an important monogenic cause of isolated early onset myopia in some series with an interesting X-linked female inheritance pattern,^69^^,^^70^ and *KDELR3*, which was identified in an exome-wide association study of very highly myopic Chinese individuals.^71^

Highly myopic children have been excluded from many clinical trials to date, despite being most at risk of sight-threatening complications.^67^ However, there are small case series reporting some promising results. In a cohort of 14 children with Mendelian myopia with a median age of 6 years and median SE of −7.5 D, the use of high dose atropine (0.5% or 1%) reduced annual axial length progression by 27% (compared to 23% in non-mendelian myopes).^72^ The use of a red light on 3 Chinese children with Sticklers syndrome has been reported – in the children, aged 3, 7, and 11 years, axial length was reported to have shortened in 5 out of the 6 eyes (by −0.07 to 0.63 mm).^73^ However, it is important to emphasize that, at present, myopia control treatments for mendelian myopia do not have an evidence base and should not be actively recommended without considerable caution and there is a need for further evidence.

## Public Health Interventions to Prevent/Reduce Incidence of Myopia

### Increasing Outdoor Time and Lighting

Given the strong protective effect of outdoor activity on myopia, there have been several public health intervention campaigns to reduce the incidence of myopia in young children. Considerable success has been reported: based on the 2010 Tian 120 program in Taiwan (increasing time outdoors to 120 minutes per day)^74^ which stabilized the rising prevalence of myopia in elementary schools (from 50% to 46%), the Yilan Myopia Prevention and Vision Improvement Program (YMVIP) started in Yilan County in Taiwan in 2014 for pre-school children aged 5 to 6 years. The prevalence of myopia fell from 15.6% and has been maintained between 8.5% and 10% over the last 8 years, even during COVID.^75^ In a recent Cochrane review examining randomized control trial evidence for the impact of outdoor activities on both incidence and progression of myopia, there was the suggestion that long‐term interventions may potentially delay myopia development but drawing conclusions was limited by the low certainty of evidence.^76^

The GOAL study was a randomized controlled trial adding 40 minutes a day of an extra outdoor lesson after school in China, involving 952 children aged 6 years from 12 schools^77^: the 3-year incidence of myopia was 40% in the control group and 30% in the intervention group; similar intervention trials in Taiwan^78^ and North East China^79^ showed a reduced incidence of myopia.

In terms of slowing progression of those children already wearing myopic correction, whereas most studies showed an effect, it was generally disappointing – for example, 1.42 cf 1.59 D in the GOAL study over 3 years.

Other myopia control programs have been instituted, predominantly education-based, for example, the Singapore National Myopia Prevention Program (NMPP),^80^ or the “20-20-2” program in the Netherlands, although there are few data regarding their success.

Novel ideas to modify the classroom environment are being investigated, including the construction of glass walled classrooms and more recently one with custom-made wallpaper covered in outdoor scenes.^81^ Forest and sky imagery with a spatial frequency spectrum comparable to outdoor environments were found to have a nominal effect in slowing down myopic shift in children who were not myopic at baseline (presented abstract, International Myopia Conference 2024).

### A Natural Experiment: COVID Lockdowns and Myopia

The COVID-19 pandemic of 2020 to 2022 provided an extreme example of the effect of an intervention in myopia, in this case, national lockdowns forcing children indoors at home. Several population-based studies in China showed a significant increase in myopia prevalence, particularly in younger children, in non-cycloplegic refraction, for example, a rise from 53% to 59% prevalence in over 800,000 children assessed between June 2019 and June 2020 in the Myopic Epidemiology and Intervention Study.^82^ Similarly Wang et al. reported a significant rise in myopia in children aged 6 to 8 years over lockdown in Feicheng, Shangdon, China,^83^ although this seemed to have resolved a year later,^84^ raising a question about whether this was truly myopia rather than accommodative spasm. Probably the best data came from the Hong Kong Children's Eye Study, which reported, after a particularly severe lockdown, again in young children in a high-myopia society, significant increases in cycloplegic-measured myopia and corresponding axial elongation. All studies showed a huge increase in screen time and many a reduction in outdoor activity in this time period.

### Atropine

Given the effectiveness of atropine to slow myopia progression, and the fact that eye growth and refractive error change is at its fastest at the onset of myopia, atropine has been suggested as a way of slowing this growth before a child is myopic. A randomized controlled trial in Hong Kong showed atropine 0.05% (and not 0.01%) was effective, reducing incidence over 2 years from 53% in the placebo group to 28.4% in the atropine 0.05% group.^85^ A comparable study in Japan examined the effect of 0.01% atropine in the prevention of myopia in a randomized controlled study and found a difference in spherical equivalent of 0.22 D at the 24-month end point between the treatment and placebo groups.^86^ This has not yet been tested in non-Asian populations, and there are considerable hurdles and a burden of proof before benefit for a prophylactic pharmacological agent would be approved by regulatory bodies.

The well-known epidemiologist Geoffrey Rose coined the term the “prevention paradox”^87^: should health promotion interventions be targeted at those who are at highest risk to lower their individual risk, or should there be a population approach to shift the distribution of risk in the population? Each has advantages and disadvantages. Where heritability explains much of the variance in a population, as in myopia, and where the whole population distribution has shifted in a myopic direction, as in many countries in East Asia, then a population approach would seem to be the correct one. However, given the distribution of refractive error is highly leptokurtotic in young populations of many countries (i.e. most children are emmetropic), and prevention of high myopia is the target (for example, those children who become myopic before the age of, say, 10 years) then a high-risk approach might be more successful.

## Technology to Aid Monitoring Myopia Progression and Risk Factors in Children

### Refractive Error

Myopia progression by regular measurement of refractive error remains the mainstay of monitoring myopia. It has been improved using standardized widefield autorefractors and cycloplegia which are widely used in epidemiological studies and clinical trials, although less so in routine clinical practice. Technology to make measurement of “true” refractive error in children simpler and easier might be helpful. A barrier to accurate measurement is the use of cycloplegic drops which for most children represent the biggest fear of an eye clinic – the invention of a “non-stinging” cycloplegic eye drop or an autorefractor where cycloplegia is truly not needed would be well-received by pediatric ophthalmologists, optometrists, and children visiting eye clinics worldwide.

### Axial Length Growth Curves

Crucially the only time myopia trajectory can be slowed is in childhood. The focus should be on minimizing eye growth to reduce the final axial length and thereby mitigate the degree of myopia and risk of sight threatening complications.^88^ Slowing myopia by 1 D should reduce the likelihood of a patient developing myopic maculopathy by 40%.^89^ Myopia progression varies with age and ethnicity^54^^,^^90^ – in meta-analysis of published data, mean axial elongation decreases as age increases (15.0% per year) and is greater in Asian children (by 27.9%) compared with non-Asians.^91^ In the United Kingdom, fast progression is generally ≥ 0.58 D per year.^92^

It is debatable whether axial length growth curves with specificity to sex, to genetic ancestry, and possibly to geographic area regardless of genetic ancestry are needed to monitor a child's axial growth compared to a similarly age-matched population normative values, or whether there should be an idealized “normal” used universally. Current normative eye growth charts can be skewed by the prevalence of myopia in that dataset, and also largely taken from cross-sectional studies rather than longitudinal studies. Normal myopic eye growth is perhaps a more reliable reference growth curve.^93^ In children of Chinese and European descent, normative growth curves for boys and girls have been developed.^54^^,^^90^ Naduvilath et al. described axial length progression in Asian children and reported children with myopia as expected had a faster progression compared to children with emmetropia, but interestingly children with incident myopia appeared to have very similar trajectories to children with myopia, and this could potentially distinguish pre-myopia in children.^94^ The trajectory of children with myopia onset under 5 years is less well understood but would be invaluable to the field.

Axial length measurement with currently available devices from several manufacturers is highly sensitive and accurate, unbiased, quick, and easy to perform. However, it is not currently widely available in many smaller optometry and non-surgical ophthalmology practices around the world. It is becoming more accessible with advances in myopia control treatments, but of course myopia control should be practiced even in the absence of axial length measurement.

### Imaging Technologies

The use of retinal photography to monitor development of myopic maculopathy has been standardized by the META-PM grading system. Monitoring of retinal photography using this grading system indicated category progression over a mean of 12.7 years in over 40% of highly myopic individuals and an increased risk of choroidal neovascularization (CNV) development with higher categories of myopic maculopathy (2.7% for category 3).^95^ Fundus photographs of 6 to 12-year-old children have been found through deep learning algorithms to independently predict risk of high myopia in teenage years, with minimal additional predictive model performance from myopia progression rates.^60^ Further work on retinal photography in children as a predictor of future complications is needed.

The choroid is a dynamic, multifunctional structure that can now be visualized by modern OCT scanning techniques. Animal models have indicated that bidirectional choroidal responses to defocus correlating with both signal and magnitude of retinal image defocus, and to precede future changes in axial length.^96^ Relevantly short-term changes in human choroidal thickness have been documented to be induced to by pharmacological, optical, and environmental stimuli – with low dose atropine incurring an increased choroidal thickness in several studies in the short-term post drop administration and long term (up to 6 months).^97^ The protective effective of time outdoors and the relationship to choroidal thickness has been less well studied but again suggests it is associated with an increase in choroidal thickness.^98^ The possibility of choroidal thickness or even volume or other measures as objective measures of myopia risk or response to therapy remains yet to be fully realized. Improving measurement accuracy and repeatability of choroidal measures with continuing advances in ocular imaging provides future promise.

The shape of the posterior globe and retina has been evaluated by magnetic resonance imaging (MRI) and more accessibly OCT. Interestingly the shape of the posterior eye may be abnormal even when the fundus appears normal with no apparent visual clues of a staphyloma. A posterior staphyloma, an out-pouching of the wall of the eye that has a radius of curvature less than the surrounding curvature, can be used to define an individual as having pathological myopia. The grading system by Curtin in 1977 based on fundus drawings has now been superseded by the information gained by OCT into types I to V.^99^ The presence of a posterior staphyloma does not contribute to a “plus” lesion on the META-PM grading system and so the implication of the grade of staphyloma on the risk of myopia, progression, myopic maculopathy, or other ocular complications is not yet clear. Relevantly staphyloma do not occur in the periphery and their position in the posterior pole suggests that associations with retinal damage and visual implications could present. Future correlation with unadjusted OCT curvature derivatives, expansion of staphyloma from a limited to extended area, and myopia related sequelae is needed.

Given the attention that has been paid to peripheral retinal defocus and the optical treatments that alter it to slow progression, further work is needed to develop reliable, robust measures of retinal shape and focus, for example, with wide-field OCT scanning and peripheral refraction devices.

### Light Exposure

Given the importance of light, further development of wearable light meters is required. Current clothing-mounted devices and wrist-worn devices^100^ have limitations, including obstruction by clothing (e.g. coats or long sleeves). Wearable light meters are generally worn for limited periods and therefore the variation between different days of the week and season may not be captured. Novel devices which can get around this issue would present significant benefits for myopia research. Spectacle-mounted devices, such as the Clouclip,^101^ resolve some of these issues but obviously require children to wear glasses, which inhibits their use in pre-myopic children.

### Reading and Screen Use

The relative contribution of reading time and screen use on myopia progression presents a measurable and modifiable risk factor which has been poorly quantified. Examples developed include a Myopia app which measures smartphone use and face-to-screen distance objectively,^36^ and the Clouclip which measures viewing distance, as does the RangeLife.^102^ Ideally, wearable sensors are required without the necessity of needing spectacle wear or limiting their use to a single screen-based device, including light-sensing and able to last all day. Emerging technologies arising from research into the impacts of screen and social media use in children may present future opportunities.

**Figure 6.**
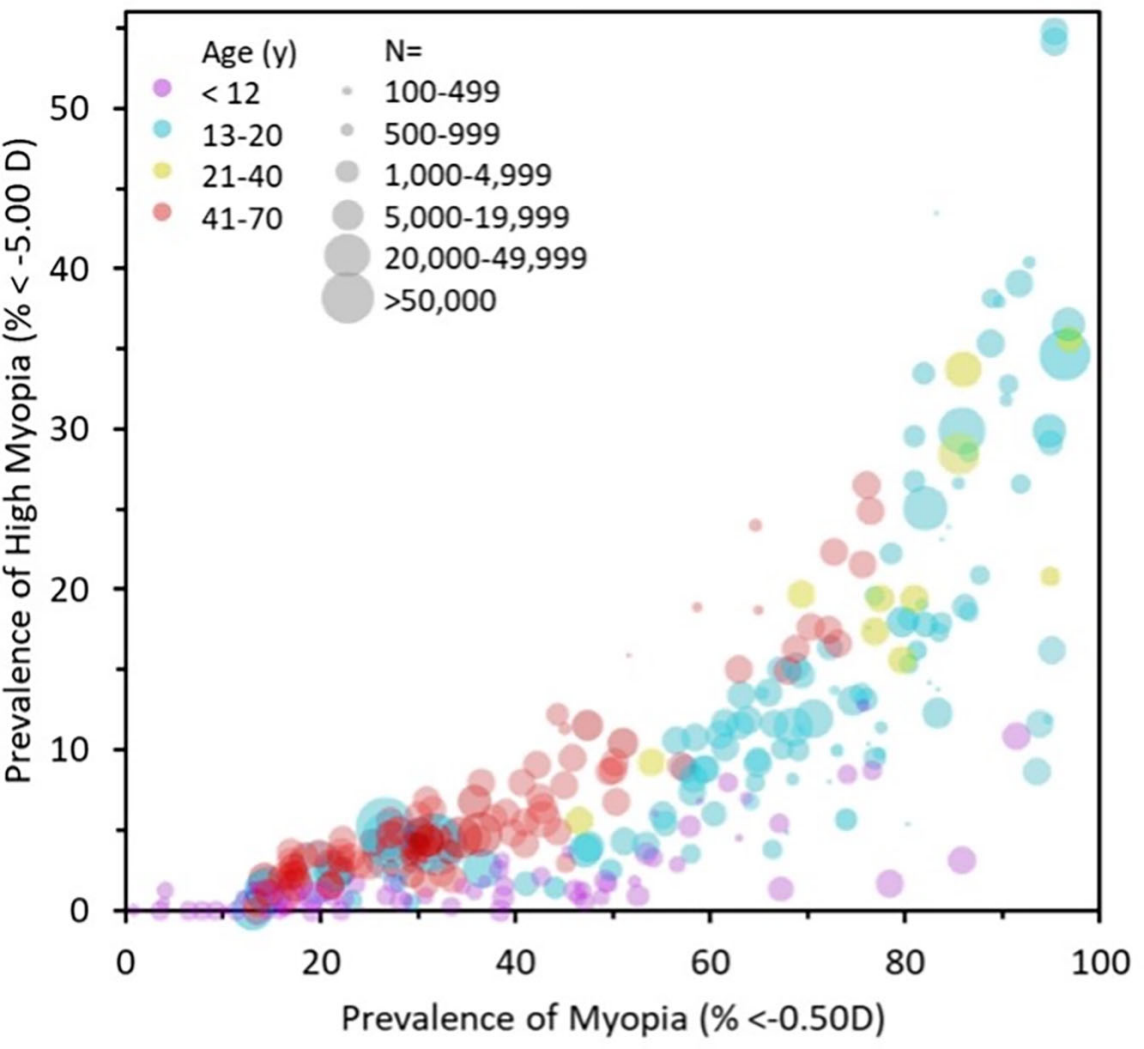
The prevalence of high myopia versus that of myopia in different age groups, with size of points indicative of the sample size of each study, from Ref. 105.

## Myopia Progression in Adulthood

Although myopia most commonly starts and progresses in childhood, a proportion of myopia develops in adulthood and certain occupational groups seem to have higher rates – a well-cited study in clinical microscopists, aged 21 to 63 years old, found 33% developed myopia after the age of 20 years.^18^ Up to a third of myopia may be of adult-onset in Western populations, less in East Asia,^103^ and whereas these myopes rarely end up with pathological myopic maculopathy, the large number of these individuals has a considerable addition to glaucoma, cataract, and retinal detachment population-attributable risk.

Progression of myopia in early adulthood occurs, particularly in the context of ongoing risk factors, such as further university education: this may average −1 D between the ages of 20 to 30 in predominantly university populations.^103^ In non-university populations, it appears progression rates may be less – in a Finnish study with 8 years of follow-up from adults in their early 20s, myopia progression averaged just under −0.5 D.^104^ In the recent IMI report,^96^ the estimated annual myopia progression rate for someone aged 30 (the mid-point age of 23 studies included) was −0.1 D per year, with higher progression rates in younger adults and slower rates in older adults.

Brennan and Bullimore presented compelling data in an analysis of 292 cohorts from 76 studies (a total of 1,034,220 individuals) that with increasing age the proportion of high myopes increases, suggesting that adult progression contributes significantly to the pathological burden of high myopia (Fig. 6).^105^ Further work is needed to explore the implications.

The pathological processes and risk factors for myopic macular degeneration are poorly understood and, at present, therapeutic options are limited. Further research in this area is needed given the strong interaction with age, the rising numbers of high myopes and increasing longevity of populations.

## Conclusions

1. The “epidemic” of myopia is likely to be a consequence of a reduction in the amount of time children are outdoors and an increase in the time they spend on close tasks, such as reading (especially in myopigenic environments where children are subject to intense focus on educational achievement).
2. Refractive error is highly heritable, meaning that in a particular environment the variance of refractive error (as opposed to the population mean) is explainable by genetic factors. This might suggest that population-level intervention is required to reduce the overall burden of myopia. Further research into genetic data and gene-environment interactions may allow future novel therapies or personalized treatments.
3. It may be that screen use is a risk factor for myopia, particularly in young children; screen use is linked to sedentary lifestyles and obesity in children, particularly in families of low SES. This might skew the demographic from myopia as a disease of well-educated children in families with relative socioeconomic advantage to one which, like so many, may also disproportionally affect children from deprived backgrounds.
4. Axial length growth curves hold significant promise as a tool to monitor risk of myopia (pre-myopia) as well as progression of myopia, as the axial elongation is the major risk factor for future vision loss. It is likely that web-based risk predictors of myopia and its progression, including other factors such as family history, additive genetic risk scores, and educational achievement, and using artificial intelligence, will be most accurate.
5. Precision or personalized medicine may be possible, given the ability to predict an individual's trajectory for refractive error to potentially prevent or slow myopia. Population-based interventions to increase time outdoors reduce incidence of myopia, but little evidence of effective personalized interventions in pre-myopic children other than generic advice. While dilute 0.05% Atropine eye drops reduced incidence of myopia in Hong Kong children, there are considerable regulatory hurdles and a burden of proof before benefit for a pharmacological intervention is possible.
6. There is evidence of some success of population-based public health interventions to reduce the burden of myopia in high-myopia environments. Given the commonality of risk factors for the other modern-day health crisis, that of childhood obesity (lack of exercise/outdoor activity and sedentary lifestyle) it would seem a combined approach to tackle these problems might seem sensible.
7. There is little longitudinal or other research into the group of children who develop high myopia at a young age, usually from known or unknown genetic causes. Although refractive error may stabilize in this group at younger ages than the more-studied “simple” myopia, it does progress in many of these children and it is not known whether myopia control treatments work in this group.
8. Progression of myopia slows in adult life, but many moderate myopes do gradually progress into the category of high myopia and likely greater complications. More evidence is needed about risk factors and if any intervention can reduce the risk.
9. Improved technologies are required to quantify outdoor exposure, continuous reading and screen duration, and working distance. Improved imaging of choroidal measures, ocular shape, and peripheral refraction with use of machine learning and other techniques is needed.

## References

[1] Dolgin E. The myopia boom. *Nature**.* 2015; 519(7543): 276–278. 25788077 10.1038/519276a

[2] Williams KM, Bertelsen G, Cumberland P, et al. Increasing prevalence of myopia in Europe and the impact of education. *Ophthalmology**.* 2015; 122(7): 1489–1497. 25983215 10.1016/j.ophtha.2015.03.018 PMC4504030

[3] Wang TJ CT, Wang TH, Lin LL, Shih YF. Changes of the ocular refraction among freshmen in National Taiwan University between 1988 and 2005. *Eye (Lond)**.* 2009; 23(5): 1168–1169. 18551136 10.1038/eye.2008.184

[4] Lin LL, Shih YF, Hsiao CK, Chen CJ, Lee LA, Hung PT. Epidemiologic study of the prevalence and severity of myopia among schoolchildren in Taiwan in 2000. *J Formos Med Assoc**.* 2001; 100(10): 684–691. 11760374

[5] Saw SM, Shankar A, Tan SB, et al. A cohort study of incident myopia in Singaporean children. *Invest Ophthalmol Vis Sci**.* 2006; 47(5): 1839–1844. 16638989 10.1167/iovs.05-1081

[6] Yotsukura E, Torii H, Inokuchi M, et al. Current prevalence of myopia and association of myopia with environmental factors among schoolchildren in Japan. *JAMA Ophthalmol**.* 2019; 137(11): 1233–1239. 31415060 10.1001/jamaophthalmol.2019.3103 PMC6696729

[7] Holden BA, Fricke TR, Wilson DA, et al. Global prevalence of myopia and high myopia and temporal trends from 2000 through 2050. *Ophthalmology**.* 2016; 123(5): 1036–1042. 26875007 10.1016/j.ophtha.2016.01.006

[8] Williams KM, Kraphol E, Yonova-Doing E, Hysi PG, Plomin R, Hammond CJ. Early life factors for myopia in the British Twins Early Development Study. *Br J Ophthalmol**.* 2019; 103(8): 1078–1084. 30401676 10.1136/bjophthalmol-2018-312439 PMC6661230

[9] Rahi JS, Cumberland PM, Peckham CS. Myopia over the lifecourse: prevalence and early life influences in the 1958 British birth cohort. *Ophthalmology**.* 2011; 118(5): 797–804. 21185080 10.1016/j.ophtha.2010.09.025

[10] Rose KA, Morgan IG, Ip J, et al. Outdoor activity reduces the prevalence of myopia in children. *Ophthalmology**.* 2008; 115(8): 1279–1285. 18294691 10.1016/j.ophtha.2007.12.019

[11] Lu B, Congdon N, Liu X, et al. Associations between near work, outdoor activity, and myopia among adolescent students in rural China: the Xichang Pediatric Refractive Error Study Report No. 2. *Arch Ophthalmol**.* 2009; 127(6): 769–775. 19506196 10.1001/archophthalmol.2009.105

[12] Lin Z, Gao TY, Vasudevan B, et al. Near work, outdoor activity, and myopia in children in rural China: the Handan Offspring Myopia Study. *BMC Ophthalmology**.* 2017; 17(1): 203. 29149871 10.1186/s12886-017-0598-9 PMC5693484

[13] Xiong S, Sankaridurg P, Naduvilath T, et al. Time spent in outdoor activities in relation to myopia prevention and control: a meta-analysis and systematic review. *Acta Ophthalmol**.* 2017; 95(6): 551–566. 28251836 10.1111/aos.13403 PMC5599950

[14] Gwiazda J, Deng L, Manny R, Norton TT, Group CS. Seasonal variations in the progression of myopia in children enrolled in the correction of myopia evaluation trial. *Invest Ophthalmol Vis Sci**.* 2014; 55(2): 752–758. 24408976 10.1167/iovs.13-13029 PMC3915767

[15] Ulaganathan S, Read SA, Collins MJ, Vincent SJ. Influence of seasons upon personal light exposure and longitudinal axial length changes in young adults. *Acta Ophthalmol**.* 2019; 97(2): e256–e265. 30288926 10.1111/aos.13904

[16] Rucker F. Monochromatic and white light and the regulation of eye growth. *Exp Eye Res**.* 2019; 184: 172–182. 31018118 10.1016/j.exer.2019.04.020 PMC6652187

[17] Torii H, Kurihara T, Seko Y, et al. Violet light exposure can be a preventive strategy against myopia progression. *EBioMedicine**.* 2017; 15: 210–219. 28063778 10.1016/j.ebiom.2016.12.007 PMC5233810

[18] Hua WJ, Jin JX, Wu XY, et al. Elevated light levels in schools have a protective effect on myopia. *Ophthalmic Physiol Opt**.* 2015; 35(3): 252–262. 25913873 10.1111/opo.12207

[19] Mirshahi A, Ponto KA, Hoehn R, et al. Myopia and level of education: results from the Gutenberg Health Study. *Ophthalmology**.* 2014; 121(10): 2047–2052. 24947658 10.1016/j.ophtha.2014.04.017

[20] Williams C, Miller LL, Gazzard G, Saw SM. A comparison of measures of reading and intelligence as risk factors for the development of myopia in a UK cohort of children. *Br J Ophthalmol**.* 2008; 92(8): 1117–1121. 18567647 10.1136/bjo.2007.128256

[21] Sherwin JC, Reacher MH, Keogh RH, Khawaja AP, Mackey DA, Foster PJ. The association between time spent outdoors and myopia in children and adolescents: a systematic review and meta-analysis. *Ophthalmology**.* 2012; 119(10): 2141–2151. 22809757 10.1016/j.ophtha.2012.04.020

[22] Morgan IG, Rose KA. Myopia and international educational performance. *Ophthalmic Physiol Opt**.* 2013; 33(3): 329–338. 23662964 10.1111/opo.12040

[23] Mountjoy E, Davies NM, Plotnikov D, et al. Education and myopia: assessing the direction of causality by mendelian randomisation. *BMJ**.* 2018; 361: k2022. 29875094 10.1136/bmj.k2022 PMC5987847

[24] French AN, Morgan IG, Mitchell P, Rose KA. Risk factors for incident myopia in Australian schoolchildren: the Sydney Adolescent Vascular and Eye Study. *Ophthalmology**.* 2013; 120(10): 2100–2108. 23672971 10.1016/j.ophtha.2013.02.035

[25] Zadnik K, Mutti DO. *Incidence and distribution of refractive anomalies**.* Philadelphia, PA: Saunders; 1998.

[26] Mutti DO, Mitchell GL, Moeschberger ML, Jones LA, Zadnik K. Parental myopia, near work, school achievement, and children's refractive error. *Invest Ophthalmol Vis Sci**.* 2002; 43(12): 3633–3640. 12454029

[27] Saw SM, Chua WH, Hong CY, et al. Nearwork in early-onset myopia. *Invest Ophthalmol Vis Sci**.* 2002; 43(2): 332–339. 11818374

[28] O'Donoghue L, Kapetanankis VV, McClelland JF, et al. Risk factors for childhood myopia: findings from the NICER study. *Invest Ophthalmol Vis Sci**.* 2015; 56(3): 1524–1530. 25655799 10.1167/iovs.14-15549

[29] Chua SY, Ikram MK, Tan CS, et al. Relative contribution of risk factors for early-onset myopia in young Asian children. *Invest Ophthalmol Vis Sci**.* 2015; 56(13): 8101–8107. 26720462 10.1167/iovs.15-16577

[30] Jones LA, Sinnott LT, Mutti DO, Mitchell GL, Moeschberger ML, Zadnik K. Parental history of myopia, sports and outdoor activities, and future myopia. *Invest Ophthalmol Vis Sci**.* 2007; 48(8): 3524–3532. 17652719 10.1167/iovs.06-1118 PMC2871403

[31] Jones-Jordan LA, Cotter SA, Kleinstein RN, et al. Time outdoors, visual activity, and myopia progression in the juvenile-onset myope. *Invest Ophthalmol Vis Sci**.* 2012; 53(11): 7169–7175. 22977132 10.1167/iovs.11-8336 PMC3474591

[32] Ip JM, Rose KA, Morgan IG, Burlutsky G, Mitchell P. Myopia and the urban environment: findings in a sample of 12-year-old Australian school children. *Invest Ophthalmol Vis Sci**.* 2008; 49(9): 3858–3863. 18469186 10.1167/iovs.07-1451

[33] Morgan IG, Wu PC, Ostrin LA, et al. IMI risk factors for myopia. *Invest Ophthalmol Vis Sci**.* 2021; 62(5): 3. 10.1167/iovs.62.5.3 PMC8083079 33909035

[34] Lanca C, Saw SM. The association between digital screen time and myopia: a systematic review. *Ophthalmic Physiol Opt**.* 2020; 40(2): 216–229. 31943280 10.1111/opo.12657

[35] Foreman J, Salim AT, Praveen A, et al. Association between digital smart device use and myopia: a systematic review and meta-analysis. *Lancet Digit Health**.* 2021; 3(12): e806–e818. 34625399 10.1016/S2589-7500(21)00135-7

[36] Enthoven CA, Polling JR, Verzijden T, et al. Smartphone use associated with refractive error in teenagers: the Myopia App Study. *Ophthalmology**.* 2021; 128(12): 1681–1688. 34245754 10.1016/j.ophtha.2021.06.016

[37] Harrington S, O'Dwyer V. The association between time spent on screens and reading with myopia, premyopia and ocular biometric and anthropometric measures in 6- to 7-year-old schoolchildren in Ireland. *Ophthalmic Physiolog Opt**.* 2023; 43(3): 505–516. 10.1111/opo.13116 36843144

[38] Zhang X, Cheung SSL, Chan HN, et al. Myopia incidence and lifestyle changes among school children during the COVID-19 pandemic: a population-based prospective study. *Br J Ophthalmol**.* 2022; 106(12): 1772–1778. 34340973 10.1136/bjophthalmol-2021-319307

[39] Aleman AC, Wang M, Schaeffel F. Reading and myopia: contrast polarity matters. *Sci Rep**.* 2018; 8(1): 10840. 30022043 10.1038/s41598-018-28904-x PMC6052140

[40] Rosner M, Laor A, Belkin M. Myopia and stature: findings in a population of 106,926 males. *Eur J Ophthalmol**.* 1995; 5(1): 1–6. 7795395 10.1177/112067219500500101

[41] Peled A, Nitzan I, Megreli J, et al. Myopia and BMI: a nationwide study of 1.3 million adolescents. *Obesity**.* 2022; 30(8): 1691–1698. 35894082 10.1002/oby.23482

[42] Lee S, Lee HJ, Lee KG, Kim J. Obesity and high myopia in children and adolescents: Korea National Health and Nutrition Examination Survey. *PLoS One**.* 2022; 17(3): e0265317. 35333875 10.1371/journal.pone.0265317 PMC8956184

[43] Tideman JWL, Polling JR, Hofman A, Jaddoe VW, Mackenbach JP, Klaver CC. Environmental factors explain socioeconomic prevalence differences in myopia in 6-year-old children. *Br J Ophthalmol**.* 2018; 102(2): 243–247. 28607175 10.1136/bjophthalmol-2017-310292

[44] Liu XN, Naduvilath TJ, Sankaridurg PR. Myopia and sleep in children-a systematic review. *Sleep**.* 2023; 46(11): zsad162. 37381700 10.1093/sleep/zsad162 PMC10639155

[45] Yuan T, Zou H. Effects of air pollution on myopia: an update on clinical evidence and biological mechanisms. *Environ Sci Pollut Res Int**.* 2022; 29(47): 70674–70685. 36031679 10.1007/s11356-022-22764-9 PMC9515022

[46] Sanfilippo PG, Hewitt AW, Hammond CJ, Mackey DA. The heritability of ocular traits. *Surv Ophthalmol**.* 2010; 55(6): 561–583. 20851442 10.1016/j.survophthal.2010.07.003

[47] Hysi PG, Choquet H, Khawaja AP, et al. Meta-analysis of 542,934 subjects of European ancestry identifies new genes and mechanisms predisposing to refractive error and myopia. *Nat Genet**.* 2020; 52(4): 401–407. 32231278 10.1038/s41588-020-0599-0 PMC7145443

[48] Tedja MS, Wojciechowski R, Hysi P, et al. Light processing and regulators are important mechanisms in refractive error. *Invest Ophthalmol Vis Sci**.* 2017; 58(8): 1225.

[49] Haarman AEG, Enthoven CA, Tedja MS, et al. Phenotypic consequences of the GJD2 risk genotype in myopia development. *Invest Ophthalmol Vis Sci**.* 2021; 62(10): 16. 10.1167/iovs.62.10.16 PMC8375003 34406332

[50] Fan Q, Guo X, Tideman JW, et al. Childhood gene-environment interactions and age-dependent effects of genetic variants associated with refractive error and myopia: the CREAM Consortium. *Sci Rep**.* 2016; 6: 25853. 27174397 10.1038/srep25853 PMC4865831

[51] Han X, Lains I, Li J, et al. Integrating genetics and metabolomics from multi-ethnic and multi-fluid data reveals putative mechanisms for age-related macular degeneration. *Cell Rep Med**.* 2023; 4(7): 101085. 37348500 10.1016/j.xcrm.2023.101085 PMC10394104

[52] Tang SM, Kam KW, French AN, et al. Independent influence of parental myopia on childhood myopia in a dose-related manner in 2,055 trios: the Hong Kong Children Eye Study. *Am J Ophthalmol**.* 2020; 218: 199–207. 32454034 10.1016/j.ajo.2020.05.026

[53] Zadnik K, Mutti DO, Friedman NE, et al. Ocular predictors of the onset of juvenile myopia. *Invest Ophthalmol Vis Sci**.* 1999; 40(9): 1936–1943. 10440246

[54] Tideman JWL, Polling JR, Vingerling JR, et al. Axial length growth and the risk of developing myopia in European children. *Acta Ophthalmol**.* 2018; 96(3): 301–309. 29265742 10.1111/aos.13603 PMC6002955

[55] Clark R, Lee SS, Du R, et al. A new polygenic score for refractive error improves detection of children at risk of high myopia but not the prediction of those at risk of myopic macular degeneration. *EBioMedicine**.* 2023; 91: 104551. 37055258 10.1016/j.ebiom.2023.104551 PMC10203044

[56] Kassam I, Foo LL, Lanca C, et al. The potential of current polygenic risk scores to predict high myopia and myopic macular degeneration in multiethnic Singapore adults. *Ophthalmology**.* 2022; 129(8): 890–902. 35358591 10.1016/j.ophtha.2022.03.022

[57] Yuan J, Qiu R, Wang Y, et al. Exome-wide genetic risk score (ExGRS) to predict high myopia across multi-ancestry populations. *Commun Med (Lond)**.* 2024; 4(1): 280. 39738800 10.1038/s43856-024-00718-1 PMC11685959

[58] Lanca C, Kassam I, Patasova K, et al. New polygenic risk score to predict high myopia in Singapore Chinese children. *Transl Vis Sci Technol**.* 2021; 10(8): 26. 10.1167/tvst.10.8.26 PMC8322707 34319387

[59] Lin HJ, Huang YT, Liao WL, et al. Developing a polygenic risk score with age and sex to identify high-risk myopia in Taiwan. *Biomedicines**.* 2024; 12(7): 1619. 39062192 10.3390/biomedicines12071619 PMC11274619

[60] Foo LL, Lim GYS, Lanca C, et al. Deep learning system to predict the 5-year risk of high myopia using fundus imaging in children. *NPJ Digit Med**.* 2023; 6(1): 10. 36702878 10.1038/s41746-023-00752-8 PMC9879938

[61] Han X, Liu C, Chen Y, He M. Myopia prediction: a systematic review. *Eye**.* 2022; 36(5): 921–929. 34645966 10.1038/s41433-021-01805-6 PMC9046389

[62] Flitcroft DI. The complex interactions of retinal, optical and environmental factors in myopia aetiology. *Prog Retin Eye Res**.* 2012; 31(6): 622–660. 22772022 10.1016/j.preteyeres.2012.06.004

[63] Haarman AEG, Enthoven CA, Tideman JWL, Tedja MS, Verhoeven VJM, Klaver CCW. The complications of myopia: a review and meta-analysis. *Invest Ophthalmol Vis Sci**.* 2020; 61(4): 49. 10.1167/iovs.61.4.49 PMC7401976 32347918

[64] Tideman JW, Snabel MC, Tedja MS, et al. Association of axial length with risk of uncorrectable visual impairment for Europeans with myopia. *JAMA Ophthalmol**.* 2016; 134(12): 1355–1363. 27768171 10.1001/jamaophthalmol.2016.4009

[65] Bikbov MM, Gilmanshin TR, Kazakbaeva GM, Panda-Jonas S, Jonas JB. Prevalence of myopic maculopathy among the very old: the Ural Very Old Study. *Invest Ophthalmol Vis Sci**.* 2024; 65(3): 29. 10.1167/iovs.65.3.29 PMC10960226 38512243

[66] Polling JR, Klaver C, Tideman JW. Myopia progression from wearing first glasses to adult age: the DREAM Study. *Br J Ophthalmol**.* 2022; 106(6): 820–824. 33495159 10.1136/bjophthalmol-2020-316234 PMC9132855

[67] Flitcroft I, Ainsworth J, Chia A, et al. IMI-management and investigation of high myopia in infants and young children. *Invest Ophthalmol Vis Sci**.* 2023; 64(6): 3. 10.1167/iovs.64.6.3 PMC10153576 37126360

[68] Haarman AEG, Thiadens A, van Tienhoven M, et al. Whole exome sequencing of known eye genes reveals genetic causes for high myopia. *Hum Mol Genet**.* 2022; 31(19): 3290–3298. 35567543 10.1093/hmg/ddac113 PMC9523556

[69] van Mazijk R, Haarman AEG, Hoefsloot LH, et al. Early onset X-linked female limited high myopia in three multigenerational families caused by novel mutations in the ARR3 gene. *Hum Mutat**.* 2022; 43(3): 380–388. 35001458 10.1002/humu.24327 PMC9303208

[70] Wang Y, Xiao X, Li X, et al. Genetic and clinical landscape of ARR3-associated MYP26: the most common cause of Mendelian early-onset high myopia with a unique inheritance. *Br J Ophthalmol**.* 2023; 107(10): 1545–1553. 36180177 10.1136/bjo-2022-321511 PMC10579186

[71] Yuan J, Zhuang YY, Liu X, et al. Exome-wide association study identifies KDELR3 mutations in extreme myopia. *Nat Commun**.* 2024; 15(1): 6703. 39112444 10.1038/s41467-024-50580-x PMC11306401

[72] van der Sande E, Polling JR, Tideman JWL, et al. Myopia control in Mendelian forms of myopia. *Ophthalmic Physiol Opt**.* 2023; 43(3): 494–504. 36882953 10.1111/opo.13115 PMC12852259

[73] Tang XH, Yu MT, Hu Y, He MG, Yang X. Axial length shortening in myopic children with Stickler syndrome after repeated low-level red-light therapy. *Int J Ophthalmol**.* 2023; 16(10): 1712–1717. 37854367 10.18240/ijo.2023.10.22 PMC10559035

[74] Wu PC, Chen CT, Chang LC, et al. Increased time outdoors is followed by reversal of the long-term trend to reduced visual acuity in Taiwan primary school students. *Ophthalmology**.* 2020; 127(11): 1462–1469. 32197911 10.1016/j.ophtha.2020.01.054

[75] Yang YC, Hsu NW, Wang CY, Shyong MP, Tsai DC. Prevalence trend of myopia after promoting eye care in preschoolers: a serial survey in Taiwan before and during the coronavirus disease 2019 pandemic. *Ophthalmology**.* 2022; 129(2): 181–190. 34425129 10.1016/j.ophtha.2021.08.013

[76] Kido A, Miyake M, Watanabe N. Interventions to increase time spent outdoors for preventing incidence and progression of myopia in children. *Cochrane Database Syst Rev**.* 2024; 6(6): CD013549. 38864362 10.1002/14651858.CD013549.pub2 PMC11167692

[77] He M, Xiang F, Zeng Y, et al. Effect of time spent outdoors at school on the development of myopia among children in China: a randomized clinical trial. *JAMA**.* 2015; 314(11): 1142–1148. 26372583 10.1001/jama.2015.10803

[78] Wu PC, Chen CT, Lin KK, et al. Myopia prevention and outdoor light intensity in a school-based cluster randomized trial. *Ophthalmology**.* 2018; 125(8): 1239–1250. 29371008 10.1016/j.ophtha.2017.12.011

[79] Jin JX, Hua WJ, Jiang X, et al. Effect of outdoor activity on myopia onset and progression in school-aged children in northeast China: the Sujiatun Eye Care Study. *BMC Ophthalmol**.* 2015; 15: 73. 26152123 10.1186/s12886-015-0052-9 PMC4495846

[80] Karuppiah V, Wong L, Tay V, Ge X, Kang LL. School-based programme to address childhood myopia in Singapore. *Singapore Med J**.* 2021; 62(2): 63–68. 31680176 10.11622/smedj.2019144 PMC8027142

[81] Yi X, Wen L, Gong Y, et al. Outdoor scene classrooms to arrest myopia: design and baseline characteristics. *Optom Vis Sci**.* 2023; 100(8): 543–549. 37499167 10.1097/OPX.0000000000002046

[82] Xu L, Ma Y, Yuan J, et al. COVID-19 quarantine reveals that behavioral changes have an effect on myopia progression. *Ophthalmology**.* 2021; 128(11): 1652–1654. 33857574 10.1016/j.ophtha.2021.04.001 PMC8463956

[83] Wang J, Li Y, Musch DC, et al. Progression of myopia in school-aged children after COVID-19 home confinement. *JAMA Ophthalmol**.* 2021; 139(3): 293–300. 33443542 10.1001/jamaophthalmol.2020.6239 PMC7809617

[84] Wang J, Han Y, Musch DC, et al. Evaluation and follow-up of myopia prevalence among school-aged children subsequent to the COVID-19 home confinement in Feicheng, China. *JAMA Ophthalmol**.* 2023; 141(4): 333–340. 36821130 10.1001/jamaophthalmol.2022.6506 PMC9951104

[85] Yam JC, Zhang XJ, Zhang Y, et al. Effect of low-concentration atropine eyedrops vs placebo on myopia incidence in children: the LAMP2 Randomized Clinical Trial. *JAMA**.* 2023; 329(6): 472–481. 36786791 10.1001/jama.2022.24162 PMC9929700

[86] Hieda O, Hiraoka T, Fujikado T, et al. Efficacy and safety of 0.01% atropine for prevention of childhood myopia in a 2-year randomized placebo-controlled study. *Jpn J Ophthalmol**.* 2021; 65(3): 315–325. 33586090 10.1007/s10384-021-00822-y

[87] Rose G. Strategy of prevention: lessons from cardiovascular disease. *Br Med J (Clin Res Ed)**.* 1981; 282(6279): 1847–1851. 10.1136/bmj.282.6279.1847 PMC1506445 6786649

[88] Wildsoet CF, Chia A, Cho P, et al. IMI - Interventions Myopia Institute: interventions for controlling myopia onset and progression report. *Invest Ophthalmol Vis Sci**.* 2019; 60(3): M106–M131. 30817829 10.1167/iovs.18-25958

[89] Bullimore MA, Brennan NA. Myopia control: why each diopter matters. *Optom Vis Sci**.* 2019; 96(6): 463–465. 31116165 10.1097/OPX.0000000000001367

[90] He X, Sankaridurg P, Naduvilath T, et al. Normative data and percentile curves for axial length and axial length/corneal curvature in Chinese children and adolescents aged 4–18 years. *Br J Ophthalmol**.* 2023; 107(2): 167–175. 34531198 10.1136/bjophthalmol-2021-319431 PMC9887397

[91] NA , Shamp W, Maynes E, Cheng X, Bullimore MA. Influence of age and race on axial elongation in myopic children: a systemic review and meta-regression. *Optum Vis Sci**.* 2024; 101(8): 497–507. 10.1097/OPX.0000000000002176 39259699

[92] Chamberlain P, Peixoto-de-Matos SC, Logan NS, Ngo C, Jones D, Young G. A 3-year randomized clinical trial of MiSight lenses for myopia control. *Optom Vis Sci**.* 2019; 96(8): 556–567. 31343513 10.1097/OPX.0000000000001410

[93] Brennan NA, Shamp W, Maynes E, Cheng X, Bullimore MA. Influence of age and race on axial elongation in myopic children: a systematic review and meta-regression. *Optom Vis Sci**.* 2024; 101(8): 497–507. 39259699 10.1097/OPX.0000000000002176

[94] Naduvilath T, He X, Xu X, Sankaridurg P. Normative data for axial elongation in Asian children. *Ophthalmic Physiol Opt**.* 2023; 43(5): 1160–1168. 37132642 10.1111/opo.13159

[95] Ohno-Matsui K, Kawasaki R, Jonas JB, et al. International photographic classification and grading system for myopic maculopathy. *Am J Ophthalmol**.* 2015; 159(5): 877–883 e877. 25634530 10.1016/j.ajo.2015.01.022

[96] Ostrin LA, Harb E, Nickla DL, et al. IMI - The dynamic choroid: new insights, challenges, and potential significance for human myopia. *Invest Ophthalmol Vis Sci**.* 2023; 64(6): 4. 10.1167/iovs.64.6.4 PMC10153586 37126359

[97] Sander BP, Collins MJ, Read SA. Short-term effect of low-dose atropine and hyperopic defocus on choroidal thickness and axial length in young myopic adults. *J Ophthalmol**.* 2019; 2019: 4782536. 31531235 10.1155/2019/4782536 PMC6721261

[98] Read SA, Pieterse EC, Alonso-Caneiro D, et al. Daily morning light therapy is associated with an increase in choroidal thickness in healthy young adults. *Sci Rep**.* 2018; 8(1): 8200. 29844529 10.1038/s41598-018-26635-7 PMC5974399

[99] Ohno-Matsui K, Lai TY, Lai CC, Cheung CM. Updates of pathologic myopia. *Prog Retin Eye Res**.* 2016; 52: 156–187. 26769165 10.1016/j.preteyeres.2015.12.001

[100] Read SA, Collins MJ, Vincent SJ. Light exposure and physical activity in myopic and emmetropic children. *Optom Vis Sci**.* 2014; 91(3): 330–341. 24413273 10.1097/OPX.0000000000000160

[101] Wen L, Cao Y, Cheng Q, et al. Objectively measured near work, outdoor exposure and myopia in children. *Br J Ophthalmol**.* 2020; 104(11): 1542–1547. 32075819 10.1136/bjophthalmol-2019-315258 PMC7587221

[102] Williams R, Bakshi S, Ostrin EJ, Ostrin LA. Continuous objective assessment of near work. *Sci Rep**.* 2019; 9(1): 6901. 31061427 10.1038/s41598-019-43408-y PMC6503122

[103] Bullimore MA, Lee SS, Schmid KL, et al. IMI-onset and progression of myopia in young adults. *Invest Ophthalmol Vis Sci**.* 2023; 64(6): 2. 10.1167/iovs.64.6.2 PMC10153577 37126362

[104] Parssinen O, Kauppinen M, Viljanen A. The progression of myopia from its onset at age 8–12 to adulthood and the influence of heredity and external factors on myopic progression. A 23-year follow-up study. *Acta Ophthalmologica**.* 2014; 92(8): 730–739. 24674576 10.1111/aos.12387

[105] Brennan NA, Cheng X, Bullimore MA. High myopia prevalence as a function of myopia prevalence and age. *Invest Ophthalmol Vis Sci**.* 2021; 62(8): 2330.

